# Association Between Metabolic Syndrome and Mild Parkinsonian Signs Progression in the Elderly

**DOI:** 10.3389/fnagi.2021.722836

**Published:** 2021-10-01

**Authors:** Zeyan Peng, Rui Zhou, Dong Liu, Min Cui, Ke Yu, Hai Yang, Ling Li, Juan Liu, Yang Chen, Wenjuan Hong, Jie Huang, Congguo Wang, Jingjing Ma, Huadong Zhou

**Affiliations:** ^1^Department of Neurology, The First Affiliated Hospital of Bengbu Medical College, Bengbu, China; ^2^Department of Neurology, Army Medical Center of PLA, Chongqing, China; ^3^Southwest Hospital, Army Medical University, Chongqing, China; ^4^State Key Laboratory of Trauma, Army Medical Center of PLA, Chongqing, China; ^5^Department of Neurology, The General Hospital of Central Theater Command, Wuhan, China; ^6^Department of Neurology, The General Hospital of Western Theater Command, Chengdu, China

**Keywords:** metabolic syndrome, mild parkinsonian signs, Parkinson's disease, progression, elderly

## Abstract

**Background:** This study investigated the impact of metabolic syndrome on the progression from mild parkinsonian signs (MPS) to Parkinson's disease (PD).

**Methods:** A total of 1,563 participants with MPS completed 6 years of follow-up. The diagnosis of metabolic syndrome was made according to Adult Treatment Panel III of the National Cholesterol Education Program. The evaluations of MPS and PD were based on the motor portion of the Unified Parkinson's Disease Rating Scale. Cox proportional hazard models were used to identify the association between metabolic syndrome and PD conversion.

**Results:** Of the 1,563 participants, 482 (30.8%) with MPS developed PD at the end of the follow-up. Metabolic syndrome (HR: 1.69, 95% CI: 1.29–2.03) was associated with the risk of PD conversion. Metabolic syndrome was associated with the progression of bradykinesia (HR: 1.85, 95% CI: 1.43–2.34), rigidity (HR: 1.36, 95% CI: 1.19–1.57), tremor (HR: 1.98, 95% CI: 1.73–2.32), and gait/balance impairment (HR: 1.66, 95% CI: 1.25–2.11). The effect of metabolic syndrome on the progression of bradykinesia and tremor was nearly two fold. Participants treated for two or three to four components of metabolic syndrome, including high blood pressure, high fasting plasma glucose, hypertriglyceridemia, and low HDL-C, had a lower risk of PD conversion.

**Conclusion:** Metabolic syndrome increased the risk of progression from MPS to PD. Participants treated for two or more components of metabolic syndrome had a lower risk of PD conversion.

## Introduction

Parkinson's disease (PD) is a rapidly developing neurodegenerative disease with a variety of etiologies and clinical manifestations. In recent years, the prevalence of PD has gradually increased worldwide (Zhang et al., 2005; Kalia and Lang, 2015; Bloem et al., 2021). In 2016, approximately 6.1 million people worldwide were affected by PD (GBD 2016 Neurology Collaborators, 2019). The prevalence rate of the disease has increased rapidly over the past 20 years. At present, the pathogenesis of PD is not yet fully understood, and no therapy can slow down or arrest the progression of PD (Bloem et al., 2021). There may be a long prodromal period before the onset of PD (Fereshtehnejad et al., 2019). Mild parkinsonian sign (MPS) is considered to be the precursor state of PD, and it includes four main symptoms: bradykinesia, rigidity, tremor, and gait and postural instability (Mahlknecht et al., 2020). The incidence rate of MPS in the elderly population is between 15–40%. The prevalence rate increases significantly with age and is associated with functional impairment and the risk of death (Wada-Isoe et al., 2016). Recent studies have found an increased risk of PD in patients with MPS (Mahlknecht et al., 2020).

Metabolic syndrome (MetS) is the aggregation of vascular risk factors in the same individual, and is associated with an increased risk of cardiovascular disease. Its main components include obesity, glucose intolerance, hypertension, hypertriglyceridemia, and low HDL-C (Eckel et al., 2005). Recent studies have shown that vascular risk factors are related to not only cardiovascular diseases, but also neurodegenerative diseases such as PD and Alzheimer's disease (Gottesman et al., 2017; Mollenhauer et al., 2019). A retrospective cohort study showed that hypertension and diabetes mellitus are associated with the risk of PD (Kummer et al., 2019). However, some studies have shown that there is no significant association between vascular risk factors and the risk of PD development (Savica et al., 2012). However, there are few studies on the relationship between MetS and progression from MPS to PD.

Whether MetS is associated with the progression from MPS to PD in the elderly in China remains unknown. Thus, we conducted a prospective study to reveal the association between MetS and MPS progression to PD.

## Methods

### Participants

We conducted an investigator-initiated, multicenter, prospective, randomized, observational study including participants with MPS, some of which had MetS. Five hospitals were randomly selected from the southern (Chongqing City, Chengdu City), central (Wuhan City), and northern (Bengbu City) regions of China. This study aimed to assess whether MetS plays a role in the conversion of MPS to PD. According to the follow-up results at the end of 6 years, the patients were divided into two groups: one consisting of patients with MPS who did not convert to PD, and the other consisting of patients with MPS who converted to PD.

**Figure 1:**
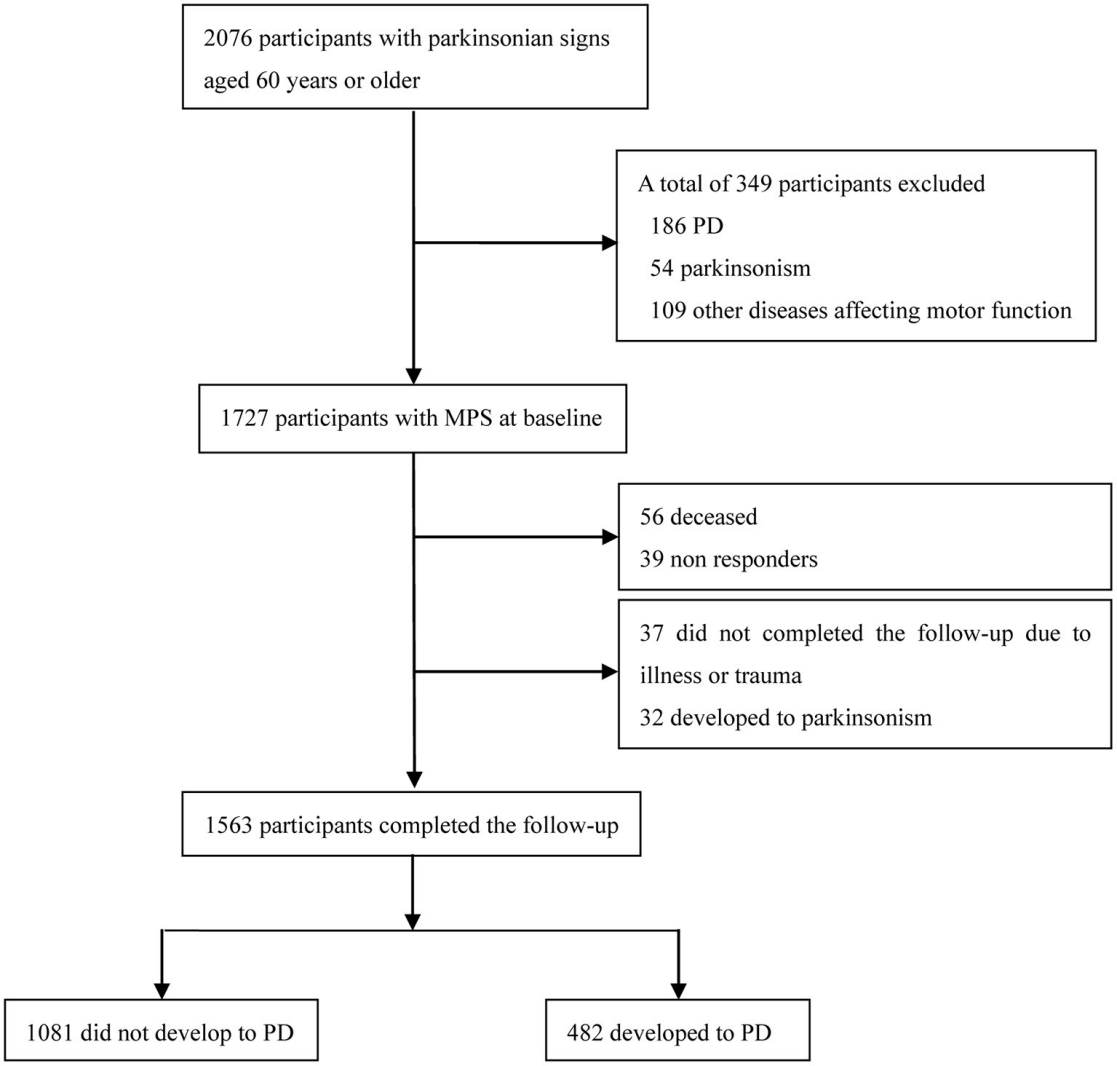
Flowchart of patient recruitment in this study. PD, Parkinson's disease; MPS, mild parkinsonian signs.

From January 2011 to December 2012, a total of 2,076 participants aged 60 years or older with parkinsonian signs were included in the study. Of the 2,076 participants, 349 did not meet the MPS diagnosis according to exclusion criteria, 1,727 with MPS were screened at baseline (567 participants from The First Affiliated Hospital of Bengbu Medical College, 342 participants from the Army Medical Center of PLA, 128 participants from Southwest Hospital, 531 participants from The General Hospital of Central Theater Command, and 159 participants from The General Hospital of Western Theater Command). A flowchart of this process is presented in Figure 1.

This study was approved by all relevant local ethics committees and research boards, and written informed consent was obtained from all participants and their caregivers. The study was funded by the National Nature Science Foundation of China, a special program for improving the scientific and technological innovation ability of Army Medical University, and the Talent Innovation Program of Bengbu Medical College.

Of the 349 participants excluded, 186 had PD, 54 had parkinsonism according to the motor portion of the Unified Parkinson's Disease Rating Scale (UPDRS-m) used to diagnose parkinsonian signs (Louis et al., 2004), and 109 had a history of head trauma. The inclusion criteria were: (1) 60 years of age or older, (2) a diagnosis of MPS, (3) long-term residents, and (4) agreement to participate in the study. The exclusion criteria were (1) parkinsonism or PD diagnosed at enrollment; (2) diseases affecting motor function, including a history of head trauma, brain tumor, epilepsy, severe stroke, encephalitis, severe arthritis, and psychiatric disorders; (3) intake of drugs that may influence parkinsonian signs, including chlorpromazine and haloperidol; (4) dementia; (5) patients who did not complete the follow-up due to diseases and trauma or developed parkinsonism during the follow-up period; and (6) participants who had moved away and non-responders. The initial evaluation time of clinical diseases was within 3 days after admission, and the MRI scan time of the subjects was within 48 h after admission.

### Demographic Data and Clinical Characteristics

Demographic data included age, sex, body mass index (BMI), and education level. The classification of current smoking and daily drinking was confirmed as previously described (Zhou et al., 2014). At baseline, 1,727 participants with MPS underwent a brain MRI (MAGNETOM Verio 3.0 T, Siemens), T1-weighted (TR/TE: 450/8.9 ms), T2-weighted (TR/TE: 5,000/87 ms), and FLAIR (TR/TE: 8,500/88 ms, inversion time: 2,000 ms) sequences. White matter hyperintensities (WMHs) were evaluated by two neurologists at every hospital who were blinded to the clinical information. BMI was calculated from the participant's height and weight. The definitions of smoking and drinking status were based on the usual habits. The diagnosis of coronary heart disease was based on the physician's diagnostic report, including myocardial infarction or stroke. Carotid artery stenosis was defined as ≥50% stenosis of the carotid artery based on validated criteria, and it was diagnosed according to the results of carotid Doppler ultrasonography (iU22 Philips Ultrasound, Bothell, WA, USA) or computed tomography angiography (CTA; Light Speed VCT 64-slice scanner; General Electric, Milwaukee, WI). The Mini-Mental State Examination (MMSE) was used to evaluate cognitive function, and the cutoff point for a diagnosis of cognitive impairment was illiteracy ≤ 17 points, elementary school ≤ 20 points, junior middle school and above ≤ 24 points (Katzman et al., 1988). Participants who did not complete the UPDRS score due to cognitive impairment were excluded.

### Diagnosis of MPS and PD

The assessment of MPS was conducted by two trained neurologists in every hospital through a standardized neurological examination, using the Unified Parkinson's Disease Rating Scale (UPDRS-m scale). The scale had a total of 27 items (each item rated from 0 to 4 points). MPS is defined as either (1) two or more items with a score of 1, or (2) one item with a score of 2 or greater (de Laat et al., 2012). Subsequently, MPS is divided into four symptoms: bradykinesia (based on nine items), rigidity (based on five items), tremor (based on seven items), and gait/balance impairment (based on six items) (de Laat et al., 2012; van der Holst et al., 2015). A symptom is considered present when participants have either (1) two or more items with a score of 1, or (2) one item with a score of 2 or greater in that symptom (de Laat et al., 2012). In addition, participants were considered to have PD if (1) they had received a diagnosis of PD by a neurologist or (2) their symptoms fulfilled the UK Parkinson's Disease Society Brain Bank diagnostic criteria (Litvan et al., 2003), or (3) both.

### Definition of MetS

The diagnosis of MetS was made according to the National Cholesterol Education Program's Adult Treatment Panel III (Eckel et al., 2005), which required the presence of at least three of the following symptoms (1) central obesity: waist circumference, >90 cm for men and >80 cm for women, according to the ethnic criteria for Asians; (2) hypertriglyceridemia: triglycerides ≥1.7 mmol/L; (3) low high density lipoprotein cholesterol: 1.0 mmol/L for men; 1.3 mmol/L for women; (4) high blood pressure: blood pressure ≥135/85 mm/Hg or medication; (5) high fasting plasma glucose: ≥6.1 mmol/L. The treatment for the components of MetS includes the treatment of high blood pressure, high fasting plasma glucose, hypertriglyceridemia, and low HDL-C, excluding central obesity.

### Medication Usage

The components of MetS that may be treated included hypertension, high blood glucose, hypertriglyceridemia, and low HDL-C, excluding central obesity. One-, two-, and three-component therapies refer to one, two, or three of the four components mentioned above. High blood pressure treatment included defined calcium channel blockers, β-blockers, diuretics, or other antihypertensive medications. High-fasting plasma glucose treatment included oral anti-hyperglycemic agents or insulin. Medications for hypertriglyceridemia treatment included statins. Medication with low HDL-C treatment included fibrates and niacins.

Some patients that converted to PD have used anti-Parkinson's medications, including levodopa-benserazide (Madopar), levodopa-carbidopa (Sinemet), pramipexole (Sifrol), piribedil (Trastal), and entacapone (Comtan).

### Follow Up

In total, 1727 participants who were enrolled in the present study participated in follow-ups for 6 years. Demographic data, medical history data, motor function and MRI images were collected at baseline. The same neurological examination for motor function was administered annually by the same neurologists.

### Statistical Analysis

In univariate analyses, normally distributed continuous variables were compared using the Student's *t*-test. Pearson's chi-square test or Fisher's exact test were used for categorical variables. Cox proportional hazard regression models were used to analyze the association between MetS and PD conversion, the association between MetS and the symptoms progression of MPS, and the association between treatment of MetS and the risk of PD conversion. Statistical analyses were performed using the SPSS software. A two-tailed *P*-value of < 0.05 was considered statistically significant.

## Results

A total of 1,727 participants with MPS were enrolled in the study at baseline. At the end of the 6-year follow-up period, a total of 164 participants were excluded, of whom 56 died, 39 could not be contacted, 37 did not complete follow-up due to illness or trauma (stroke, dementia, trauma, psychiatric disorders, severe organ dysfunction), and 32 developed parkinsonism. Finally, 1,563 (90.5%) participants with MPS completed the follow-up and were included in the statistical analysis. Among the 1,563 participants, 1,081 (69.2%) did not develop PD (where 42 reverted to normal and 1039 remained with MPS), and 482 (30.8%) developed PD.

### Baseline Characteristics of Participants With PD Conversion

The demographic factors and clinical characteristics of the participants with PD conversion at baseline are shown in Table 1. Participants that developed PD were significantly older, had more WMHs, had higher fasting plasma glucose levels, compared with those who did not develop PD (*P* < 0.05). The proportion of daily drinking was higher in those who developed PD, but the proportion of current smokers was lower (*P* < 0.05). Moreover, there was a significant difference in the prevalence of MetS between MPS participants who developed PD and those who did not develop PD (*P* < 0.001).

**Table 1:** Baseline characteristics of participants with PD conversion.

	**PD free (*n* = 1,081)**	**PD conversion** **(*n* = 482)**	***P*** **-value**
Age, mean (SD), years	66.0 (7.9)	66.9 (7.5)	0.016
Male, *n* (%)	539 (49.9)	244 (50.6)	0.781
BMI, mean (SD)	24.2 (3.7)	24.5 (3.6)	0.066
Lower educational level (<6 y), *n* (%)	369 (34.1)	161 (33.4)	0.778
Family history of PD	93 (8.6)	47 (9.8)	0.463
**Smoking status**			
Never smoking *n* (%)	442 (40.9)	211 (43.8)	0.285
Past smoking *n* (%)	235 (21.7)	118 (24.5)	0.231
Current smoking *n* (%)	404 (37.4)	153 (31.7)	0.032
**Drinking status**			
Occasional drinking *n* (%)	398 (36.8)	164 (34.0)	0.288
Monthly drinking *n* (%)	183 (16.9)	79 (16.4)	0.792
Weekly drinking *n* (%)	159 (14.7)	61 (12.7)	0.281
Daily drinking *n* (%)	341 (31.5)	178 (36.9)	0.037
WMHs, *n* (%)	371 (34.3)	195 (40.5)	0.020
Lacunar infarction/TIA	294 (27.2)	152 (31.5)	0.079
Coronary heart disease	265 (24.5)	139 (28.8)	0.071
COPD	138 (12.8)	76 (15.8)	0.111
Coronary artery disease, *n* (%)	372 (34.4)	148 (30.7)	0.151
Carotid arterial stenosis (≥50%), *n* (%)	226 (20.9)	117 (24.3)	0.137
Systolic BP, mean (SD), mmHg	142.5 (13.7)	143.9 (14.2)	0.069
Diastolic BP, mean (SD), mmHg	90.6 (6.3)	91.3 (6.8)	0.055
Fasting plasma glucose, mean (SD), mmol/l	5.7 (1.7)	6.0 (2.4)	0.013
Total cholesterol, mean (SD), mean (SD), mmol/l	4.3 (2.6)	4.5 (2.7)	0.086
Triglycerides mean, mean (SD), mean (SD), mmol/l	1.5 (1.1)	1.6 (1.2)	0.060
LDL-C, mean (SD), mean (SD), mmol/l	2.6 (1.6)	2.7 (1.5)	0.117
HDL-C, mean (SD), mean (SD), mmol/l	1.5 (1.2)	1.4 (1.1)	0.054
MetS, *n* (%)	278 (25.7)	181 (37.6)	<0.001
Each component of MetS			
Central obesity, *n* (%)	382 (35.3)	175 (36.2)	0.775
High blood pressure, *n* (%)	430 (39.8)	217 (45.0)	0.052
High fasting plasma glucose, *n* (%)	409 (37.8)	214 (44.4)	0.014
Hypertriglyceridemia, *n* (%)	376 (34.8)	190 (39.4)	0.073
Low HDL-C, *n* (%)	338 (31.3)	169 (35.1)	0.139
Number of MetS components, *n* (%)			
0	328 (30.3)	114 (23.7)	0.007
1	277 (25.6)	101 (20.9)	0.046
2	198 (18.3)	86 (17.8)	0.822
3	151 (14.0)	89 (18.5)	0.023
4-5	127 (11.7)	92 (19.1)	<0.001
UPDRS part III score, mean (SD)	1.6 (1.2)	1.7 (1.3)	0.075
MMSE, mean (SD)	27.1 (3.8)	27.4 (3.5)	0.064
ADL, mean (SD)	64.4 (8.1)	63.8 (7.9)	0.085

*ADL, Activities of Daily Living; BMI, body mass index; HDL-C, high density lipoprotein cholesterol; LDL-C, low density lipoprotein cholesterol; MetS, metabolic syndrome; MMSE, Mini-Mental State Examination; PD, Parkinson's disease; SD, standard deviation; TIA: Transient ischemic attack; WMHs, white matter hyperintensities. The meaning of grey value P < 0.05*.

### Association of MetS With PD Conversion

The association between MetS and PD conversion is shown in Table 2. After adjustment for age, daily drinking, current smoking, WMHs, and MetS, age (HR: 1.30, 95% CI: 1.09–1.54), current smoking (HR: 0.81, 95% CI: 0.66–0.99), daily drinking (HR: 1.25, 95% CI: 1.01–1.50), and WMHs (HR: 1.28, 95% CI: 1.02–1.59) were associated with the risk of PD conversion. In addition, MetS (HR: 1.69, 95% CI: 1.29–2.03) and high fasting plasma glucose levels (HR: 1.29, 95% CI: 1.03–1.60) were associated with the risk of PD conversion. The number of MetS components showed that three components (HR: 1.39, 95% CI: 1.04–1.84) and four to five components (HR: 1.75, 95% CI: 1.30–2.36) were associated with the risk of PD conversion.

**Table 2:** Association of baseline characteristics with PD conversion.

	**Unadjusted HR (95% CI)**	**Adjusted HR (95% CI)** ^&^
Age	1.34 (1.12–1.63)^**^	1.30 (1.09–1.54)^**^
Current smoking	0.78 (0.62–0.98)^*^	0.81 (0.66–0.99)^*^
Daily drinking	1.27 (1.02–1.59)^*^	1.25 (1.01–1.50)^*^
WMHs	1.30 (1.04–1.62)^**^	1.28 (1.02–1.59)^*^
MetS	1.74 (1.38–2.19)^**^	1.69 (1.29–2.03)^**^
Central obesity	1.04 (0.83–1.30)	1.02 (0.81–1.27)
High blood pressure	1.24 (0.99–1.54)	1.23 (0.97–1.51)
High fasting plasma glucose	1.31 (1.05–1.63)^*^	1.29 (1.03–1.60)^*^
Hypertriglyceridemia	1.22 (0.98–1.52)	1.20 (0.96–1.50)
Low HDL-C	1.19 (0.95–1.45)	1.16 (0.92–1.41)
**Number of MetS components**		
0		
1	0.79 (0.61–1.02)	0.81 (0.63–1.01)
2	0.97 (0.73–1.28)	0.98 (0.75–1.26)
3	1.40 (1.05–1.86)^*^	1.39 (1.04–1.84)^*^
4-5	1.77 (1.32–2.38)^**^	1.75 (1.30–2.36)^**^

*BMI, body mass index; CI, confidence interval; HDL-C, high density lipoprotein cholesterol; HR, hazard ratio; LDL-C, low density lipoprotein cholesterol; MetS, metabolic syndrome; PD, Parkinson's disease; WMHs, white matter hyperintensities*.

& *The model is adjusted for age, daily drinking, current smoking, metabolic syndrome, and WMHs*.

* *P < 0.05;*

** *P < 0.01*.

### MetS and Symptom Progression of MPS at End of the 6 Year Follow-Up

Participants with an increased score (≥1 point) for each individual symptom during follow-up were considered to have progressed. The association between MetS and symptom progression of MPS was analyzed using the Cox proportional hazard regression (Table 3). After adjustment for potential factors, MetS was associated with the progression of bradykinesia (HR: 1.85, 95% CI: 1.43–2.34), rigidity (HR: 1.36, 95% CI: 1.19–1.57), tremor (HR: 1.98, 95% CI: 1.73–2.32) and gait/balance impairment (HR: 1.66, 95% CI: 1.25–2.11). This suggests that MetS has the greatest impact on the progression of tremor (nearly twice as much) and the least impact on rigidity. The association between the components of MetS and MPS progression was further analyzed. High blood pressure was associated with the progression of bradykinesia (HR: 1.29, 95% CI: 1.10–1.58) and tremor (HR: 1.31, 95% CI: 1.12–1.65), and high fasting plasma glucose was associated with the progression of bradykinesia (HR: 1.28, 95% CI: 1.07–1.55), rigidity (HR: 1.26, 95% CI: 1.04–1.52), tremor (HR: 1.40, 95% CI: 1.12–1.69) and gait/balance impairment (HR: 1.17, 95% CI: 1.02–1.49).

**Table 3:** MetS with symptom progression of MPS.

	**Bradykinesia adjusted HR (95% CI)** ^&^	**Rigidity adjusted HR (95% CI)** ^&^	**Tremor adjusted HR (95% CI)** ^&^	**Gait/balance adjusted HR (95% CI)** ^&^
**MetS**	1.85 (1.43–2.34)^**^	1.36 (1.19–1.57)^*^	1.98 (1.73–2.32)^**^	1.66 (1.25–2.11)^**^
Central obesity	1.04 (0.91–1.15)	1.01 (0.90–1.12)	1.06 (0.93–1.16)	1.03 (0.89–1.17)
High blood pressure	1.29 (1.10–1.58)^*^	1.18 (0.89–1.22)	1.31 (1.12–1.65)^*^	1.22 (0.95–1.34)
High fasting plasma glucose	1.28 (1.07–1.55)^*^	1.26 (1.04–1.52)^*^	1.40 (1.12–1.69)^*^	1.17 (1.02–1.49)^*^
Hypertriglyceridemia	1.14 (1.01–1.48)	1.08 (0.93–1.38)	1.17 (1.06–1.51)	1.05 (0.95–1.41)
Low HDL-C	1.07 (0.95–1.43)	1.04 (0.91–1.35)	1.15 (0.98–1.47)	1.12 (0.93–1.28)
**Number of MetS components**				
1 component	1.06 (0.93–1.16)	1.04 (0.90–1.14)	1.02 (0.88–1.12)	1.08 (0.96–1.19)
2 components	1.17 (0.96–1.29)	1.06 (0.91–1.22)	1.13 (0.89–1.19)	1.15 (0.94–1.22)
3 components	1.54 (1.19–1.95)^*^	1.17 (0.98–1.52)	1.64 (1.28–2.06)^*^	1.46 (1.12–2.01)^*^
4-5 components	1.99 (1.61–2.56)^**^	1.45 (1.18–1.93)^**^	2.24 (1.63–2.89)^**^	2.05 (1.82–2.64)^**^

*CI, confidence interval; HDL-C, high density lipoprotein cholesterol; HR, hazard ratio; MetS, metabolic syndrome; WMHs, white matter hyperintensities*.

& *The model is adjusted for age, daily drinking, current smoking, metabolic syndrome, and WMHs*.

* *P < 0.05;*

** *P < 0.01*.

In addition, the association between the number of MetS components and symptom progression of MPS was analyzed. The presence of three components of MetS was associated with the progression of bradykinesia (HR: 1.54, 95% CI: 1.19–1.95), tremor (HR: 1.64, 95% CI: 1.28–2.06) and gait/balance impairment (HR: 1.46, 95% CI: 1.12–2.01). The presence of four or five components of MetS was associated with the progression of bradykinesia (HR: 1.99, 95% CI: 1.61–2.56), rigidity (HR: 1.45, 95% CI: 1.18–1.93), tremor (HR: 2.24, 95% CI: 1.63–2.89), and gait/balance impairment (HR: 2.05, 95% CI: 1.82–2.64).

### Incidence of MPS Conversion During Follow-Up

**Figure 2:**
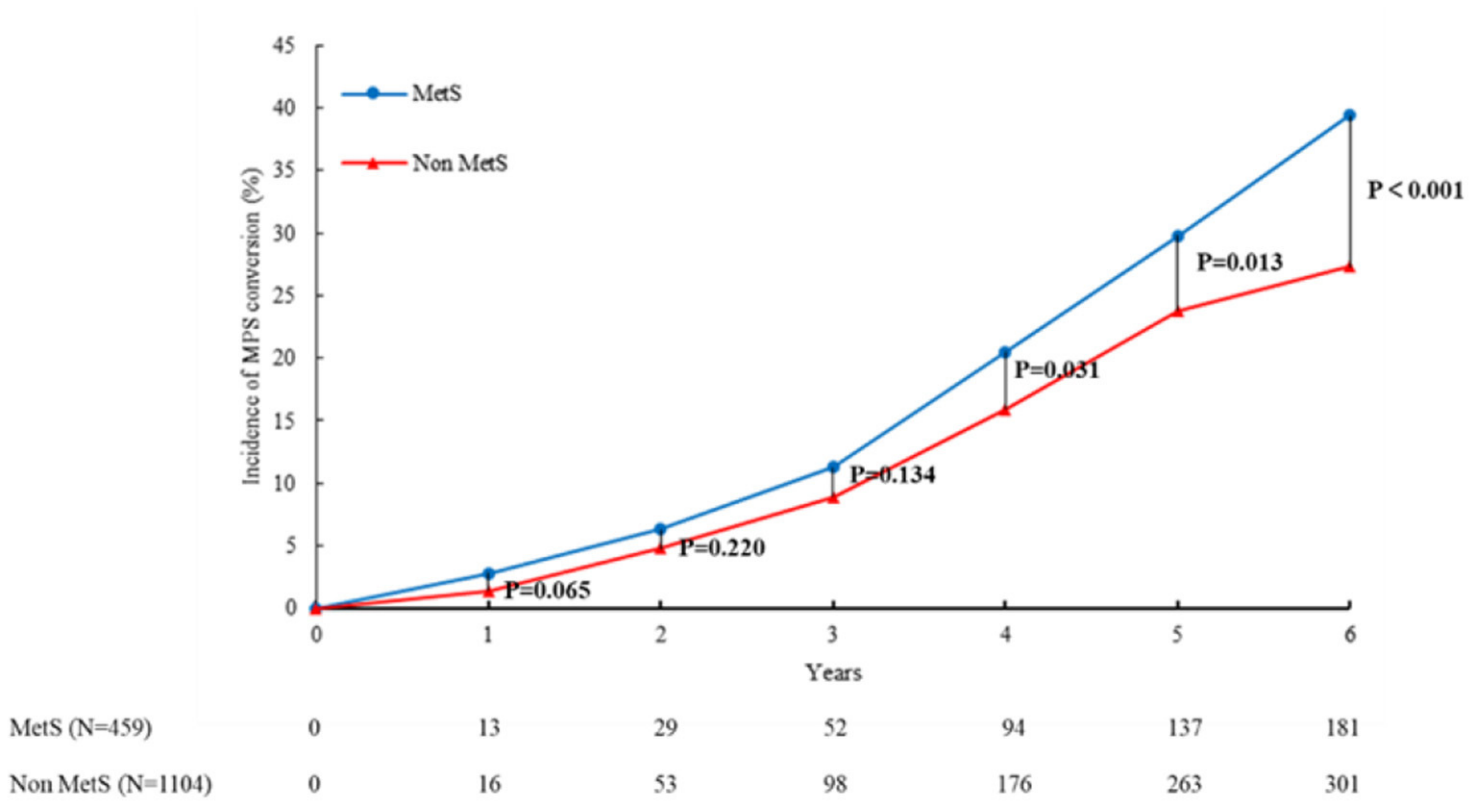
MetS and PD conversion by age. The incidence of MPS conversion in subjects with and without MetS across the years of follow-up is shown in this figure. The incidence of MPS conversion in subjects with MetS was higher than that in subjects without MetS during the follow-up of 6 years. The incidence of MPS conversion in subjects with MetS was significantly higher than that in subjects without MetS, particularly in the 4th, 5th, and 6th years (*p* < 0.05). MetS, metabolic syndrome; MPS, mild parkinsonian signs.

The incidence of MPS conversion in subjects with and without MetS across the years of follow-up is shown in Figure 2. The incidence of MPS conversion during the 6-year follow-up in subjects with MetS was higher than that in subjects without MetS. The incidence of MPS conversion in subjects with MetS was significantly higher than that in subjects without MetS, particularly in the 4th, 5th, and 6th years (*p* < 0.05).

### MetS and PD Conversion by Age

**Figure 3:**
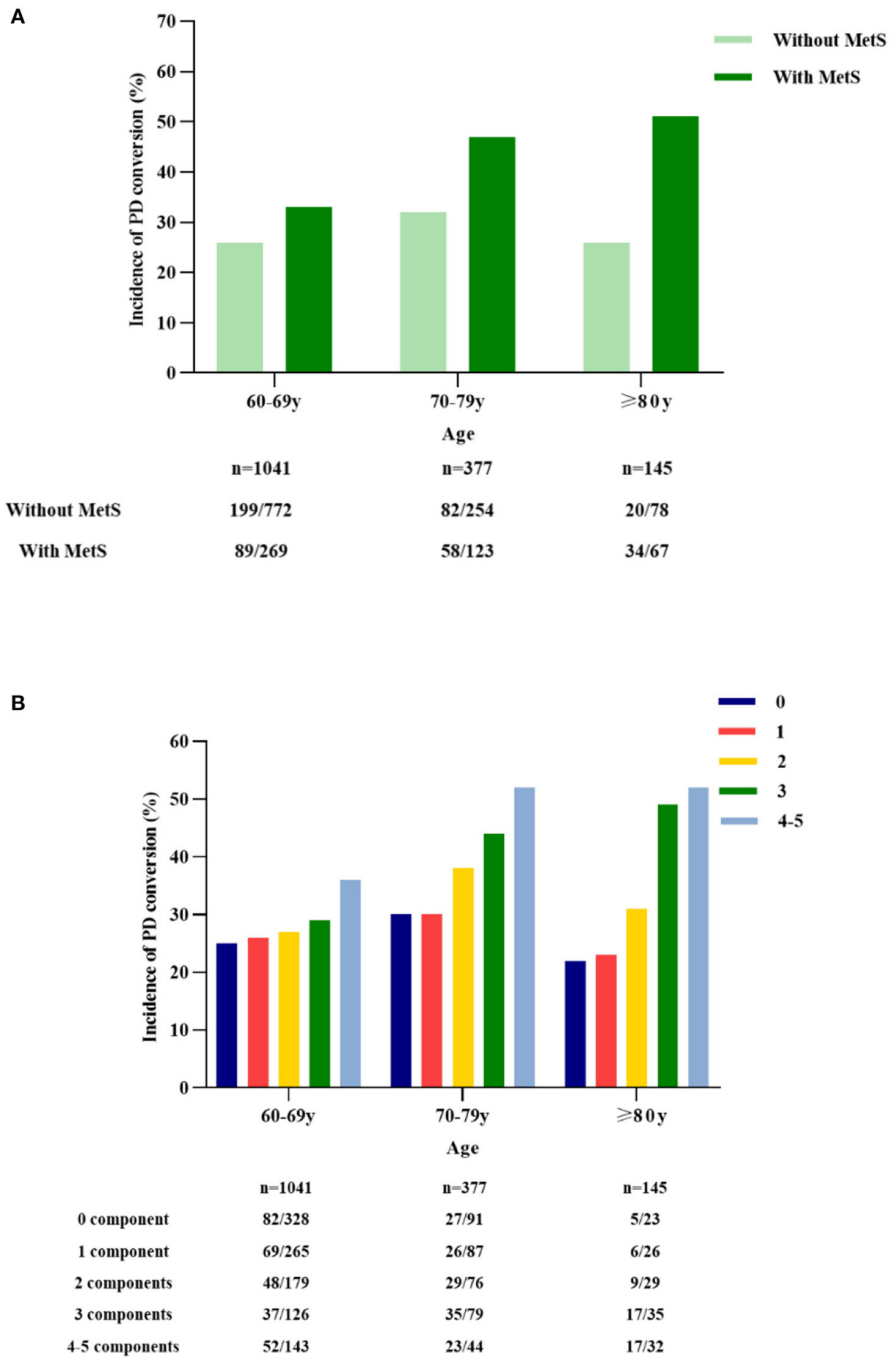
MetS and incidence of PD conversion by age. The participants were divided into three groups according to age: 60–69 years old, 70–79 years old, and 80 years and older. The incidence of PD conversion was significantly higher in participants with metabolic syndrome than in those with non-metabolic syndrome in the 60–69, 70–79, and 80 years and older age groups (*P* < 0.05; *P* < 0.01; **A**). The incidence of PD conversion was analyzed according to the number of MetS components. Compared with the non-metabolic syndrome component, the incidence of PD conversion was significantly higher in participants with three and four to five metabolic syndrome components in the 70–79 and 80 years and older groups (*P* < 0.05), and the incidence of PD conversion was significantly higher in participants with four to five metabolic syndrome components in the 60–69 years old group (*P* < 0.05; **B**). PD, Parkinson's disease; MetS, metabolic syndrome.

According to age, the participants were divided into three groups: 60–69 years old, 70–79 years old, and 80 years and older. The incidence of PD conversion was significantly higher in participants with MetS than in those with non-metabolic syndrome in the 60–69, 70–79 and 80 and older age groups (*P* < 0.05 and *P* < 0.01, respectively) (Figure 3). We further analyzed the incidence of PD conversion according to the number of MetS components. Compared with the non-metabolic syndrome component, the incidence of PD conversion was significantly higher in participants with three or four to five components of MetS in the 70–79 years old group and 80 and older age groups (*P* < 0.05), and the incidence of PD conversion was significantly higher in participants with four to five components of MetS in the 60–69 old age group (*P* < 0.05).

### Association Between MetS Treatment and PD Conversion

The association between medical treatment of MetS components and PD conversion was analyzed using a Cox regression analysis (Table 4). After adjustment for potential factors, treatment for three to four components of MetS (HR: 0.62, 95% CI: 0.45–0.97) or two components (HR: 0.68, 95% CI: 0.48–0.99) was associated with a lower risk of PD conversion compared with no treatment for MetS.

**Table 4:** Treatment of MetS with risk of PD conversion.

	**PD free** ***n* = 278**	**PD conversion** ***n* = 181**	**Adjusted HR (95% CI)^&^**
**Treatment of MetS components^#^**			
3-4 components, *n* (%)	72 (25.9)	31 (17.1)	0.62 (0.45–0.97)^*^
2 components, *n* (%)	103 (37.1)	50 (27.6)	0.68 (0.48–0.99)^*^
1 component, *n* (%)	71 (25.5)	53 (29.3)	1.18 (0.72–1.68)
None treatment, *n* (%)	32 (11.5)	47 (26.0)	2.70 (1.64–4.43)^**^

*CI, confidence interval; HDL-C, high density lipoprotein cholesterol; HR, hazard ratio; MetS, metabolic syndrome; PD, Parkinson's disease*.

& *The model is adjusted for age, daily drinking, current smoking, metabolic syndrome, and WMHs*.

# *Patients with metabolic syndrome were treated with high blood pressure, high fasting plasma glucose, hypertriglyceridemia, and low HDL-C, excluding central obesity. Three-component therapy, two-component therapy, and one-component therapy refer to three, two or one of the four components of high blood pressure, high fasting plasma glucose, hypertriglyceridemia, and low HDL-C*.

* *P < 0.05;*

** *P < 0.01*.

## Discussion

In this prospective study of Chinese elderly population, we found that participants with MetS had an approximately, 1.7-fold increased risk of progression from MPS to PD, and the incidence of PD conversion increased with the increase in the number of MetS components compared with participants with non-metabolic syndrome. It was further found that MetS increased the risk of major symptom progression of PD, including bradykinesia, rigidity, tremor and gait/balance impairment. Additionally, we also found that the interventions for MetS components may have reduced the risk of PD conversion.

At present, there are few reports on the relationship between MetS and the risk of progression from MPS to PD, but there are some studies on the relationship between vascular risk factors and MPS or PD (Louis and Luchsinger, 2006; Hatate et al., 2016). One study revealed participants with MPS were more likely to have diabetes mellitus, heart disease and stroke compared to those without MPS (Louis and Luchsinger, 2006). Logistic regression analysis revealed that the number of vascular diseases was related to MPS. The combination of diabetes mellitus and heart disease increased the risk of MPS by 70%. The presence of MPS in the elderly might partly reflect the accumulation of vascular pathological changes in the white matter area caused by preventable vascular diseases. A study showed that PD patients had higher Framingham risk score and axial motion scores (Kotagal et al., 2014). Vascular risk factors may be related to the axial motor function of PD. A meta-analysis based on the population cohort studies suggested that the risk of PD in patients with hypertension was 1.70 compared to non-hypertensive patients (95% CI: 1.60–1.80) (Chen et al., 2019). A Korean study that included 33,443 participants with PD during a 7.3-year follow-up period showed that diabetes mellitus was associated with an increased risk of PD (Jeong et al., 2020). A study in Taiwan that retrospectively assessed the risk of PD among patients with diabetes showed that diabetes mellitus was significantly associated with the risk of PD (Sun et al., 2012). A large prospective study showed that subjects aged 25–54 years with high total cholesterol levels had an increased risk of PD development (Hu et al., 2008). In an average 11.5-year follow-up of 503,497 middle-aged participants in China, 603 participants were diagnosed with PD (Kizza et al., 2019). Obesity has been shown to be associated with a high risk of PD in this population. However, a retrospective study in the United States did not support the association between cholesterol levels, hypertension or diabetes and the progression of PD (Savica et al., 2012). A Japanese case-control study found that hypertension, hypercholesterolemia, and diabetes mellitus were significantly associated with a decreased risk of PD, with adjusted ORs of were 0.43 (95% CI: 0.29–0.64), 0.58 (95% CI: 0.33–0.97), and 0.38 (95% CI: 0.17–0.79), respectively (Miyake et al., 2010). The subjects in the above studies were from China, Japan, Korea, the United States, and Finland. The results showed that diabetes, hypertension, dyslipidemia, and obesity might increase the risk of MPS or PD, but the results are controversial. These studies did not discuss the above vascular risk factors as a whole (such as MetS), and these studies only discussed the impact of vascular risk factors on the risk of MPS or PD. In contrast, in our study, we explored the effect of MetS on the risk of conversion to PD.

These studies suggest that MetS is related to the development of PD. A multicenter, double-blind study involving PD participants showed that the total UPDRS score of participants with MetS increased over time compared to participants with non-MetS, mainly due to the increase in exercise score (Leehey et al., 2017). Another large cohort study in South Korea found that people with MetS had a higher risk of PD than those with non-metabolic syndrome. Each component of MetS was positively correlated with the risk of PD, including obesity, hypertension, hyperglycemia, hypertriglyceridemia and low HDL-C. There was a positive association between the incidence of PD and MetS scores, and the incidence of PD increased with the increase in the MetS scores (Nam et al., 2018). In addition, this result of a retrospective cohort study with an average follow-up of 5.2 years showed that hypertension, diabetes mellitus, and smoking were associated with the subsequent diagnosis of PD (Kummer et al., 2019). The symptoms of early PD that progressed in 4 years with arterial hypertension and dysglycemia were identified as dangerous signs of faster disease PD progression (Mollenhauer et al., 2019). These studies mainly discussed the impact of MetS on the progressive risk of PD, while our study mainly discussed the impact of MetS on MPS as a precursor to progression to PD.

MetS might affect the progression of MPS to PD through the following mechanisms. A previous study showed that the progression of MPS might be caused by dysfunction of the nigra striatum system. Substantia nigra hyperechogenicity was associated with an increased risk of MPS in the general elderly population (Mahlknecht et al., 2015). A study on neurodegeneration after cerebral ischemia suggested that hypertension, diabetes mellitus, and dyslipidemia led to pathological changes in the cerebral arteries and arterioles, and that cerebral ischemia, the substantia nigra and the dopaminergic neurons of ischemic areas could be damaged (Rodriguez-Grande et al., 2013). On the other hand, cerebral blood flow shortfalls are early findings in neurodegenerative disorders in humans and animal models (Sweeney et al., 2018). A previous study that examined the role of cerebral blood flow and the blood-brain barrier in the pathogenesis of PD suggested a common pathway linking the cerebral vascular contributions to neurodegeneration. Some evidence has shown that hyperglycemia is one of the causes of neurodegenerative changes involving the nigral striatum pathway, which is the neuroanatomical basis of motor symptoms in patients with PD (Sergi et al., 2019). Dopaminergic neurons in the substantia nigra are susceptible to oxidative stress, which represents the consequences of oxidative stress caused by hyperglycemia. Furthermore, the formation of advanced glycation end products plays an important role in regulating the harmful effects of hyperglycemia on dopaminergic neurons. Another study showed that hypercholesterolemia in a mouse model of PD resulted in the loss of dopamine neurons in the substantia nigra and a reduction in dopamine levels in the striatum (Paul et al., 2017). Hypercholesterolemia leads to oxidative stress in the striatum nigra, which aggravats oxidative stress in the PD mouse model. In addition, dyslipidemia induces an inflammatory response in the nigrostriatal pathway and aggravates the loss of dopaminergic neurons. The inflammatory response has been demonstrated to be the cause of neuron loss (Sliter et al., 2018). Secretion of proinflammatory cytokines in the serum of patients with PD was elevated, including IL-6, TNF, IL-1β, and IFN-γ. The loss of dopaminergic neurons suggests that inflammation promotes the development of the PD phenotype.

There may be a long prodromal period before PD becomes clinically observable. At present, the establishment of prodromal symptoms has no clinical significance other than symptom suppression, although the identification of prodromal PD may have an impact when treatments become available. Currently, there is no treatment that can slow or stop the progression of PD. However, owing to the new understanding of the cause and mechanism of neuronal death, several promising strategies have been proposed to test the potential of treating PD (Bloem et al., 2021).

This study showed that MetS is a risk factor for PD conversion. However, the pathogenesis of PD conversion is not yet fully understood. It has been proven to be an age-related neurodegenerative disease (Bloem et al., 2021) with a genetic component related to the known PD genes. Other causal relationships include a family history of PD, which doubles the risk of PD (Bloem et al., 2021). There are many studies on the relationship between PD and smoking, and most of them suggest that smoking can reduce the incidence of PD (Kalia and Lang, 2015). Some studies have shown that alcohol consumption may increase the risk of PD (Brighina et al., 2009; Paul et al., 2019). Our previous studies also suggested an association between WMH and PD. In a 5-year follow-up study of 636 MPS patients, severe WMH was associated with PD conversion (HR: 2.69, 95% CI: 1.63–4.51; Huo et al., 2020). On the other hand, in the past 6 months, there has been concern that COVID-19 will increase the risk of PD (Brundin et al., 2020; Cohen et al., 2020; Merello et al., 2021). Hypoosmia is a common feature of patients with COVID-19 and PD. The relationship between COVID-19 and PD requires further investigation.

This study has several limitations. First, our study was limited to a cohort of Han Chinese participants with MPS. Due to this selection bias, our results may not be generalizable to other ethnicities and cohorts. Future studies including more local and ethnic participants are needed to reduce this selection bias. Second, we did not study the relationship between MetS and the progression of MPS to parkinsonism, and the relationship between MetS and the progression of MPS to vascular parkinsonism. Evaluating the relationship between MetS and MPS progression will be more conducive to understanding MPS progression.

In this study, we found that MetS was significantly associated with the progression of MPS to PD and with the progression of motor disorders in MPS. Interventions for MetS could reduce the risk of MPS progression.

## Data Availability Statement

The original contributions presented in the study are included in the article/supplementary material, further inquiries can be directed to the corresponding author.

## Ethics Statement

The studies involving human participants were reviewed and approved by the Ethics Committee of Army Medical Center of PLA. The subjects received oral and written information regarding the study and provided written informed consent to participate in this study.

## Author Contributions

ZP: conceptualization, data curation, investigation, resources, visualization, and writing of the original draft. RZ: data curation, investigation, methodology, and reviewing and editing. YC: methodology, software, and visualization. MC and KY: data curation, investigation, and resources. WH: investigation, resources, and visualization. JH: investigation, resources, and software. DL: investigation, methodology, and software. JL: formal analysis and methodology. CW and JM: data curation and investigation. HY: methodology, resources, software, and visualization. LL: conceptualization, methodology, and project administration. HZ: conceptualization, funding acquisition, project administration, validation, and writing-review and editing. All authors contributed to the article and approved the submitted version.

## Funding

This work was funded by (1) the National Nature Science Foundation of China (81771177); (2) the special program for improving scientific and technological innovation ability of Army Medical University (20191102); and (3) Talent Innovation Program of Bengbu Medical College (BBMC 20190013).

## Conflict of Interest

The authors declare that the research was conducted in the absence of any commercial or financial relationships that could be construed as a potential conflict of interest.

## Publisher's Note

All claims expressed in this article are solely those of the authors and do not necessarily represent those of their affiliated organizations, or those of the publisher, the editors and the reviewers. Any product that may be evaluated in this article, or claim that may be made by its manufacturer, is not guaranteed or endorsed by the publisher.
