# Corrigendum: Association Between Metabolic Syndrome and Mild Parkinsonian Signs Progression in the Elderly

**DOI:** 10.3389/fnagi.2021.790099

**Published:** 2021-11-24

**Authors:** Zeyan Peng, Rui Zhou, Dong Liu, Min Cui, Ke Yu, Hai Yang, Ling Li, Juan Liu, Yang Chen, Wenjuan Hong, Jie Huang, Congguo Wang, Jingjing Ma, Huadong Zhou

**Affiliations:** ^1^Department of Neurology, The First Affiliated Hospital of Bengbu Medical College, Bengbu, China; ^2^Department of Neurology, Army Medical Center of PLA, Chongqing, China; ^3^Southwest Hospital, Army Medical University, Chongqing, China; ^4^State Key Laboratory of Trauma, Army Medical Center of PLA, Chongqing, China; ^5^Department of Neurology, The General Hospital of Central Theater Command, Wuhan, China; ^6^Department of Neurology, The General Hospital of Western Theater Command, Chengdu, China

**Keywords:** metabolic syndrome, mild parkinsonian signs, Parkinson's disease, progression, elderly

In the published article, there was an error in the affiliations of all the authors. Instead of:

Zeyan Peng^1^, Rui Zhou^2^, Dong Liu^3^, Min Cui^4^, Ke Yu^5^, Hai Yang^1^, Ling Li^1^, Juan Liu^1^, Yang Chen^1^, Wenjuan Hong^6^, Jie Huang^6^, Congguo Wang^6^, Jingjing Ma^6^ and Huadong Zhou^6^*

^1^ Department of Neurology, The First Affiliated Hospital of Bengbu Medical College, Bengbu, China

^2^ Department of Neurology, Army Medical Center of PLA, Chongqing, China

^3^ Southwest Hospital, Army Medical University, Chongqing, China

^4^ State Key Laboratory of Trauma, Army Medical Center of PLA, Chongqing, China

^5^ Department of Neurology, The General Hospital of Central Theater Command, Wuhan, China

^6^ Department of Neurology, The General Hospital of Western Theater Command, Chengdu, China

It should be:

Zeyan Peng^1, 2^, Rui Zhou^3^, Dong Liu^4^, Min Cui^5^, Ke Yu^6^, Hai Yang^2^, Ling Li^2^, Juan Liu^2^, Yang Chen^2^, Wenjuan Hong^1^, Jie Huang^1^, Congguo Wang^1^, Jingjing Ma^1^, Huadong Zhou^1*^

^1^Department of Neurology, The First Affiliated Hospital of Bengbu Medical College, Bengbu, China

^2^ Department of Neurology, Army Medical Center of PLA, Chongqing, China

^3^ Southwest Hospital, Army Medical University, Chongqing, China

^4^ State Key Laboratory of Trauma, Army Medical Center of PLA, Chongqing, China

^5^ Department of Neurology, The General Hospital of Central Theater Command, Wuhan, China

^6^ Department of Neurology, The General Hospital of Western Theater Command, Chengdu, China

The authors apologize for this error and state that this does not change the scientific conclusions of the article in any way. The original article has been updated.

## Publisher's Note

All claims expressed in this article are solely those of the authors and do not necessarily represent those of their affiliated organizations, or those of the publisher, the editors and the reviewers. Any product that may be evaluated in this article, or claim that may be made by its manufacturer, is not guaranteed or endorsed by the publisher.

